# Meta-Analysis of the rs4779584 Polymorphism and Colorectal Cancer Risk

**DOI:** 10.1371/journal.pone.0089736

**Published:** 2014-02-24

**Authors:** Hua Yang, Ya Gao, Tian Feng, Tian-Bo Jin, Long-Li Kang, Chao Chen

**Affiliations:** 1 School of Life Sciences, Northwest University, Xi’an, Shaanxi, China; 2 School of Medicine, Xi’an Jiaotong University, Xi’an, China; 3 National Engineering Research Center for Miniaturized Detection Systems, Xi’an, Shaanxi, China; 4 Key Laboratory of High Altitude Environment and Genes Related to Diseases of Tibet Autonomous Region, School of Medicine, Tibet University for Nationalities, Xianyang, Shaanxi, China; Duke Cancer Institute, United States of America

## Abstract

**Purpose:**

Several researchers have suggested that the rs4779584 (15q13.3) polymorphism is associated with an increased risk of developing colorectal cancer (CRC). However, past results remain inconclusive. We addressed this controversy by performing a meta-analysis of the relationship between rs4779584 of *GREM1-SCG5* and colorectal cancer.

**Methods:**

We selected 12 case-control studies involving 11,769 cases of CRC and 14,328 healthy controls. The association between the rs4779584 polymorphism and CRC was examined by the overall odds ratio (OR) with a 95% confidence interval (CI). We used different genetic model analyses, sensitivity analyses, and assessments of bias in our meta-analysis.

**Results:**

*GREM1-SCG5* rs4779584 polymorphisms were associated with CRC in all of the genetic models that were examined in this meta-analysis of 12 case-control studies.

**Conclusion:**

*GREM1-SCG5* rs4779584 polymorphisms may increase the risk of developing colorectal cancer.

## Introduction

Colorectal cancer (CRC) is the third most common malignancy and the fourth most common cause of cancer-related death in the world [1]. The incidence of CRC is increasing each year [2], [3]. Although CRC is a heterogeneous disease, patients with advanced stages of disease generally have a poor prognosis [4]. The development of CRC is influenced by lifestyle and dietary factors. In addition, the prognosis of patients with CRC is largely affected by genetic components [5]–[7]. Current data suggests that the rs4779584 polymorphism within the 15q13.3 chromosomal region is associated with an increased risk of developing colorectal cancer [8]–[16]. Rs4779584 lies between the *GREM1* and *SCG5* genes. *GREM1* encodes gremlin 1, which is a signaling molecule in the transforming growth factor-β (TGF-β) pathway. TGF-β signaling has been implicated in tumor invasion and metastasis [17]. *SCG5* encodes secretogranin V, which is an important neuroendocrine signaling molecule that appears to influence cellular proliferation in the large bowel based on nutrient availability or systemic hormonal effects [18].

Several research groups have reported associations between this single-nucleotide polymorphism (SNP) and the risk of CRC. However, because single studies are often underpowered due to inadequate sample sizes, the results from past studies are inconclusive. Thus, we performed a meta-analysis to more precisely characterize the association between the rs4779584 polymorphism and colorectal cancer.

## Materials and Methods

### 2.1 Search Strategy and Selection Criteria

We searched five electronic databases (PubMed, Medline, Web of Knowledge, CNKI, and Google Scholar) to identify eligible studies that were published before September 2013. Articles were sought with the following key words: “colorectal cancer”, “rs4779584”, “polymorphism”, and “SCG5 or GREM1”. We also supplemented this search by reviewing the reference lists of all of the retrieved publications and identifying additional relevant articles.

The following inclusion criteria were used to identify articles for our meta-analysis. (1) The study involved unrelated individuals. (2) Sufficient genotype data were presented to allow calculation of the odds ratios (ORs). (3) The study clearly described the diagnosis of CRC and the sources of the cases and controls. (4) The genotype distribution complied with the Hardy–Weinberg equilibrium (HWE). In addition, we excluded reviews and redundant studies. Finally, we selected 11 available studies, which involved 11,769 cases of CRC and 14,328 healthy controls.

### 2.2 Data Extraction

Two investigators used a standardized form to independently extract data to improve the reliability of our results. The following information was extracted from each study: first author, publication year, ethnicity (country), source of controls, number of cases and controls, and the genotype frequencies of the cases and controls.

### 2.3 Statistical Analyses

All analyses were performed with the STATA Version 11.0 software (Stata Corp, College Station, TX). All P values in this study were two-sided, and P = 0.05 was set as the threshold value for statistical significance. To evaluate associations between rs4779584 polymorphisms and risk of CRC, the pooled odds ratio (OR) and associated 95% confidence interval (CI) were calculated. We used the following models to calculate different ORs: the allele model (A vs. a), the additive genetic model (AA vs. aa), the dominant genetic model (AA+Aa vs. aa), and the recessive genetic model (AA vs. Aa+aa). If the P value was greater than 0.100 according to the Q-test, indicating a lack of heterogeneity among studies, the summary OR estimate of each study was calculated by a fixed-effects model (the Mantel–Haenszel method). Otherwise, the random-effects model (the DerSimonian-Laird method) was performed. Heterogeneity was estimated with the Cochran’s Q-statistic, and P<0.05 was considered to be an indication of statistically significant heterogeneity [19]. We also quantified the effect of heterogeneity with the I^2^ test [20]. As a guide, I^2^ values ranged from 0 to 100%, and values of 25%, 50%, and 75% were considered to represent low, moderate, and high levels of heterogeneity, respectively. The funnel plot was drawn to assess publication biases. The test suggested was used to test the funnel-plot symmetry. This test involved building a regression model, in which the standardized estimate of the size effect was the dependent variable, and the inverse of the standard error was the independent variable. If the intercept was significantly different from zero, the estimate of the effect was considered biased. The significance of the pooled OR was determined with the Z test. Each study was removed in turn for sensitivity analyses, and the remaining studies were reanalyzed to assess the stability of results.

## Results

### 3.1 Characteristics of the Included Studies

**Figure 1:**
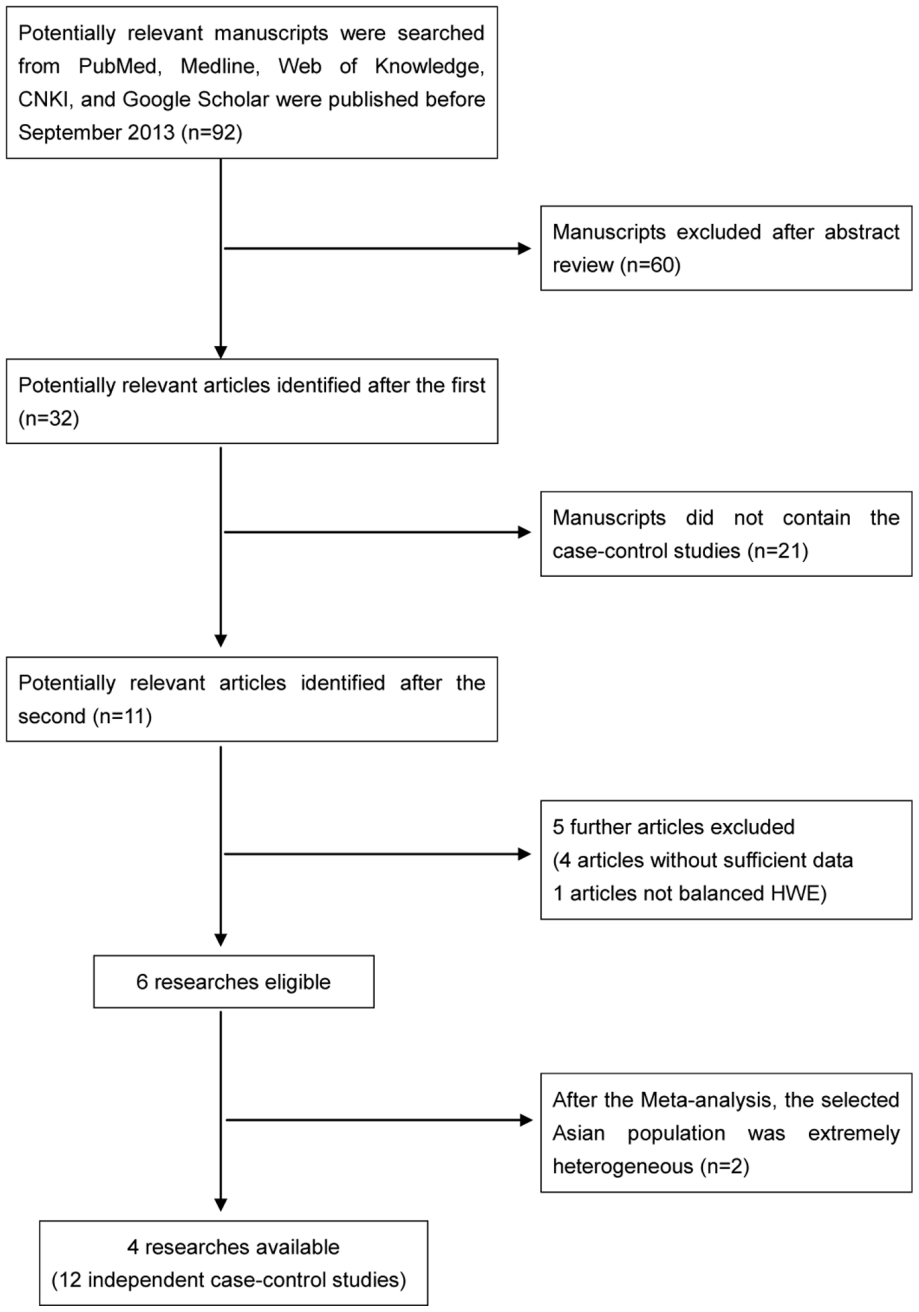
Flow diagram of study selection.

According to search, we identified 92 potentially relevant articles. On the basis of the abstract, 32 studies were reviewed in their entirety. During the extraction of data, 21 articles were removed, because of these articles did not contain the case-control studies. Among the 11 papers, four papers without sufficient data and one article that did not fit the HWE during calculations. Finally, we selected 6 researches eligible. After our meta-analysis found that the selected Asian population was extremely heterogeneous. Finally we selected 12 independent case-control studies from 4 articles involved 11,769 cases of CRC and 14,328 healthy controls in our meta-analysis. The characteristics of the studies are listed in Table 1. Flow diagram of study selection in Figure 1.

**Table 1:** Characteristics of studies included in the meta-analysis.

ID	Study	year	Population	Source of controls	Ethnic group	Case/control	HWE *p*
1	Xiong et al. [16]	2010	Chinese	PB	Asian	2124/2124	0.076
2	Ho et al. [10]	2011	Hong Kong Chinese	HB	Asian	716/714	0.763
3	Jaeger et al. [7]	2008	British	PB	Caucasian	730/960	0.726
4	Houlston et al.^ a^ [29]	2008	British	HB	Caucasian	922/929	0.577
5	Houlston et al.^b^	2008	Scottish	HB	Caucasian	922/930	0.209
6	Houlston et al.^c^	2008	British	HB	Caucasian	922/931	0.796
7	Houlston et al.^d^	2008	Scottish	HB	Caucasian	922/932	0.799
8	Talseth-Palmer et al. [30]	2010	British	PB	Caucasian	258/313	0.969
9	Tomlinson et al.^e^ [11]	2011	Australian	HB	Caucasian	591/2353	0.877
10	Tomlinson et al.^f^	2011	Spanish	HB	Caucasian	1410/1410	0.266
11	Tomlinson et al. ^g^	2011	American, Canadian, Australian	HB	Caucasian	1332/1084	0.333
12	Tomlinson et al.^h^	2011	Finnish	HB	Caucasian	988/864	0.671
13	Tomlinson et al.^i^	2011	British	HB	Caucasian	621/1121	0.324
14	Tomlinson et al.^j^	2011	British	HB	Caucasian	2151/2501	0.822

HB: hospital-based; PB: population-based.

### 3.2 Meta-analysis Databases

**Figure 2:**
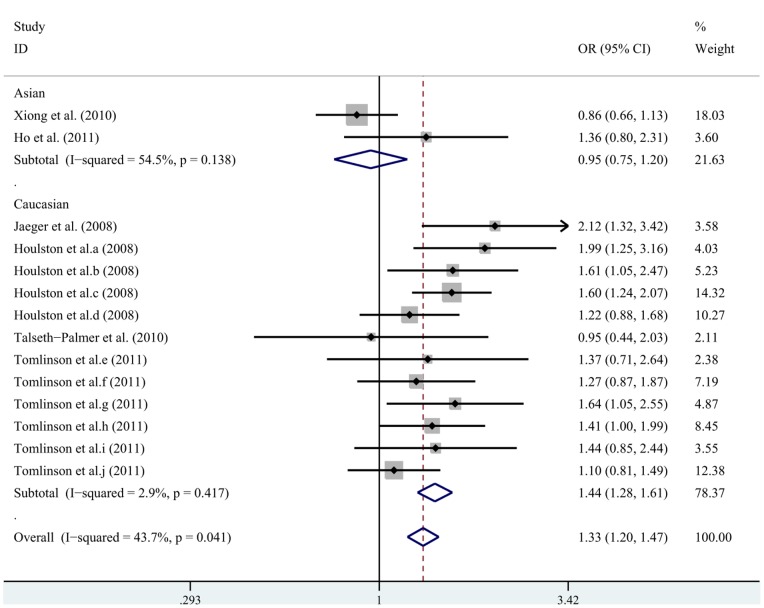
Forest plots for the rs4779584 polymorphism and risk of CRC in the additive genetic model.

Different genetic models were used in our analysis (Table 2). After pooling all of the selected studies into the meta-analysis, we found that the rs4779584 polymorphism was significantly associated with an increased risk of CRC in Caucasian subjects. The following data were obtained: in the additive model (TT vs. CC; OR = 1.44, 95% CI: 1.28–1.61; TT vs. CT; OR = 1.29, 95% CI: 1.15–1.45); in the dominant model (TT/TC vs. CC; OR = 1.15, 95% CI: 1.07–1.24); in the recessive model (TT vs. CC/CT; OR = 1.38, 95% CI: 1.24–1.55). Forest plots for rs4779584 in the additive genetic models are shown in Figure 2.

**Table 2:** Meta-analysis of the rs4779584 polymorphism.

geneticmodel		OR (95% CI)			Z			P		
		Caucasian	Chinese	Hong-KongChinese	Caucasian	Chinese	Hong-KongChinese	Caucasian	Chinese	Hong-KongChinese
additive	TT vs. CC	1.44(1.28–1.61)	0.86(0.66–1.13)	1.36(0.80–2.31)	6.30	1.07	1.12	<0.001	0.283	0.262
	TT vs. CT	1.29(1.15–1.45)	1.10(0.97–1.26)	1.32(1.05–1.66)	4.29	1.47	2.36	<0.001	0.142	0.018
dominant	TT/TC vs. CC	1.15(1.07–1.24)	0.84(0.64–1.09)	1.25(0.73–2.11)	3.94	1.33	0.81	<0.001	0.184	0.416
recessive	TT vs. CC/CT	1.38(1.24–1.55)	1.06(0.94–1.21)	1.32(1.06–1.65)	5.72	0.96	2.49	<0.001	0.336	0.013

### 3.3 Test of Heterogeneity

Significant heterogeneity existed in all of the genetic models (Table 3). The selected Asian population was extremely heterogeneous. Thus, we did not include the Asian population in our meta-analysis. However, we did compare the OR of the Asian population with that of the European population.

**Table 3:** Degree of heterogeneity in meta-analyses of the rs4779584 polymorphism.

genetic model		Heterogeneity statistic			P			I- squared (%)		
		Asian	Caucasian	overall	Asian	Caucasian	overall	Asian	Caucasian	overall
additive	TT vs. CC	2.20	11.32	23.09	0.138	0.417	0.041	54.5	2.9	43.7
	TT vs. CT	1.72	5.36	8.84	0.190	0.912	0.785	41.9	0.0	0.0
dominant	TT/TC vs. CC	1.74	21.50	27.01	0.187	0.029	0.012	42.7	48.8	51.9
recessive	TT vs. CC/CT	2.86	8.81	18.52	0.091	0.639	0.139	65.0	0.0	29.8

### 3.4 Bias Diagnostics

**Figure 3:**
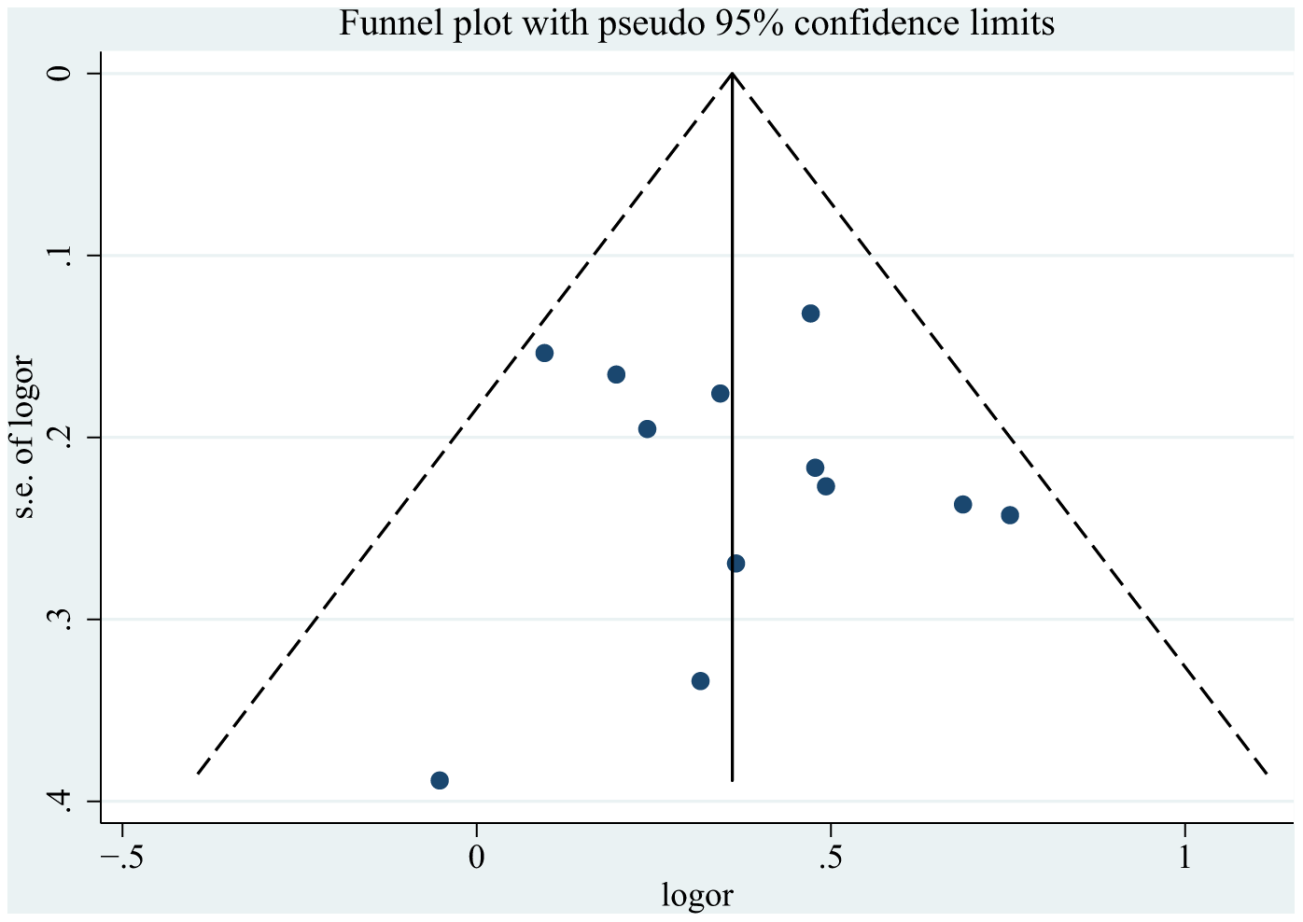
Funnel plot of the meta-analysis of CRC risk and the rs4779584 polymorphism.

Begger’s funnel plot and Egger’s linear regression test were performed to assess the publication biases of the selected studies. The shape of the funnel plot for publication bias appeared to be symmetrical, although there was some uncertainty regarding the degree of symmetry (Figure 3). The estimate of the effect was considered to be biased. In the recessive model (TT vs. CC/CT), the results from the Begger’s test (P = 0.45) and Egger’s linear regression test (t = 0.42, P = 0.682) did not show evidence of publication bias. Furthermore, the 95% confidence interval (95% CI: −1.73–2.54) included zero, indicating a lack of publication bias. Other genetic models also suggested a lack of publication bias.

### 3.5 Sensitivity Analyses

**Figure 4:**
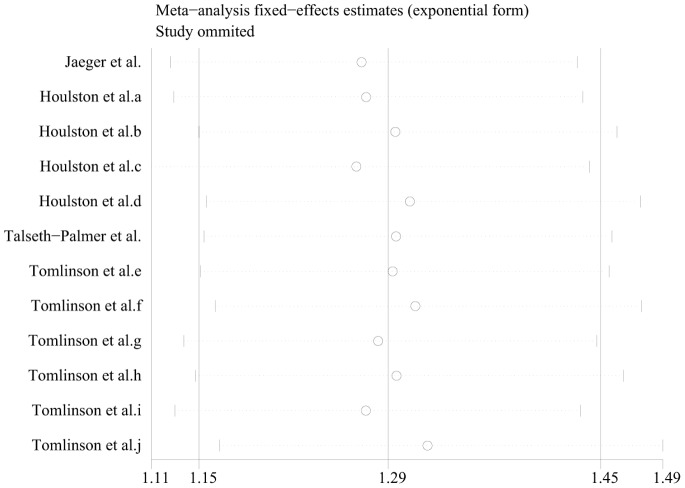
Sensitivity analysis of the odds ratio coefficients among Caucasian subject.

Sensitivity analyses were performed after sequentially removing each eligible study. This approach is regarded as an indispensable step for analyzing multiple criteria. The significance of the pooled ORs was not influenced by any single study in the recessive genetic model (Figure 4), indicating that our results were statistically robust.

## Discussion

A growing number of studies suggest that the rs4779584 polymorphism is associated with an increased susceptibility to colorectal cancer. However, the results of past studies have been controversial, with some studies supporting a significant association, whereas others refute this association. In the present study, to confirm that the rs4779584 polymorphism plays a role in the development of CRC, we conducted a meta-analysis of 12 independent case-control studies, which included 11,769cases and 14,328 controls. The major finding of the present meta-analysis was that 15q13.3 rs4779584 was a risk factor for CRC in the Caucasian population. In comparison to previous meta-analyses, our analysis included a greater number of studies. Therefore, a larger sample size and increased statistical power were obtained. Moreover, the present meta-analysis included an acceptable quality evaluation system, minimizing the potential for bias.

A statistically significant level of heterogeneity was found in the Asian population but not in Caucasians. Thus, we eliminated the Asian population (Chinese and Hong-Kong Chinese) from our meta-analysis. In addition, data in HapMap (http://www.hapmap.org/) demonstrated that T was a minor allele in the Caucasian population, whereas it is a major allele in the Asian population. Different allelic frequencies in different ethnic groups may account for these discrepancies. Heterogeneity may be due to many factors, such as differences in the characteristics of controls, diverse genotyping methods, small sample size, and a mixed population from different geographic regions.

Rs4779584 is located between *GREM1* and *SCG5*. Jaeger et al. [9] were the first to report that *GREM1*-*SCG5* was strongly associated with an increased risk for CRC (for rs4779584, P = 4.44×10^−14^). Although the functional relevance of *GREM1* rs4779584 is not completely understood, studies suggest that *GREM1* maps to human chromosome 15q13-q15, specifically at 15q13.3 [21]. Gremlin1 (*GREM1*) is a bone morphogenetic protein (BMP) antagonist and putative angiogenesis-modulating protein. *GREM1* is silenced by promoter hypermethylation in human malignancies [22]. In the colon, *GREM1* is one of several BMP antagonists produced by sub-epithelial myofibroblasts. *GREM1* binds to and inactivates the ligands BMP2 and BMP4, which are primarily produced by inter-cryptal stromal cells [13]. Gremlin 1 is a signaling component of the TGF-β pathway, which suppresses cellular proliferation and modulates cell invasion, immune regulation, and the tumor microenvironment [23]. It is generally accepted that excessive production and/or activation of TGF-β by tumor cells promotes cancer progression by mechanisms that include increased tumor neoangiogenesis, extracellular matrix production, upregulation of proteases, and inhibition of immune surveillance in the cancer host [24], [25]. The TGF-β/BMP pathway plays an important role in colorectal tumorigenesis [26], [27]. In particular, *GREM1* initiates and maintains important developmental and disease-associated activities. Thus, *GREM1* may increase tumor proliferation through stromal effects [28]. Although *SCG5* is genetically and functionally a less critical candidate than *GREM1*, neuroendocrine signaling involving SCG5 may influence cellular proliferation in the large bowel based on nutrient availability or systemic hormonal effects [18].

There were some limitations in our meta-analysis. First, only published studies were included in the present meta-analysis. Second, our meta-analysis was based on unadjusted ORs estimates, because not all of the studies reported adjusted ORs. In addition, in cases in which adjusted ORs were presented, the ORs were not adjusted by the same potential confounders, such as ethnicity, age, gender, or geographic distribution. Finally, our meta-analysis focused on one population (the Caucasian population). We excluded the Asian population due to a relatively small sample size and significant heterogeneity.

## Conclusion

In summary, this meta-analysis provides reliable evidence that the *GREM1-SCG5* rs4779584 polymorphism may be a risk factor for CRC among Caucasian subjects. Moreover, future investigations into the combined effects of genes and the environment may improve current understanding of the associations between *GREM1-SCG5* rs4779584 and the risk of developing colorectal cancer. Further work will help clarify the clinical and biological implications of these associations.

## Supporting Information

File S1 PRISMA 2009 Checklist.
(DOC)
